# The role of orexin A in the pathogenesis of ischaemic stroke at high altitude

**DOI:** 10.1113/EP092314

**Published:** 2025-05-31

**Authors:** Minxia Zhu, Xu Guo, Jing Guo, Zhaojun Zhang, Kexin Zhang, Yaqi Lei, Yuhe Wang, Haoshuang Zhan

**Affiliations:** ^1^ Key Laboratory of High Altitude Hypoxia Environment and Life Health, School of Medicine Xizang Minzu University Xianyang Shaanxi China; ^2^ Affiliated Hospital of Xizang Minzu University Xianyang Shaanxi China

**Keywords:** cerebral ischaemia–reperfusion injury, high altitude, ischaemic stroke, orexin A

## Abstract

Orexin offers protection against cerebral ischaemia–reperfusion injury, with high altitude playing a key role in modulating its expression. This study aimed to investigate the effect of high altitude on orexin expression and its pathophysiological mechanisms involved in high altitude stroke injury. In this study, changes of orexin expression were observed by simulating hypoxia at different altitudes, and the changes of orexin and its receptor were analysed by constructing a middle cerebral artery occlusion (MCAO) model after high altitude simulation. Finally, the protective effect of orexin on cerebral ischaemia–reperfusion injury was evaluated by exogenous orexin intervention. The results indicated that at an altitude of 4000 m, orexin expression was increased, but then decreased at higher altitudes. Rats exposed to 4000 m hypoxia simulation and MCAO exhibited increased orexin and type 1 receptor expression. Exogenous orexin A administration reduced infarct size, improved microcirculation blood flow in the ischaemic cortex, accelerated blood flow, elevated blood oxygen saturation and mitigated systemic oxidative stress and inflammation. These findings confirm our hypothesis that 4000 m altitude promotes orexin expression, thereby attenuating cerebral ischaemia–reperfusion injury through enhanced microcirculation, reduced oxidative stress and inflammation.

## Abstract (highlights)

- **What is the central question of this study?**
  What is the effects of high altitude on orexin expression and what are its pathophysiological mechanisms involved in high altitude stroke injury?
- **What is the main finding and its importance?**
  4000 m altitude promotes orexin expression, thereby attenuating cerebral ischaemia–reperfusion injury through enhanced microcirculation and reduced oxidative stress and inflammation.

## Graphical abstract

Schematic illustration of the experimental design. This study investigated the role of Orexin A in the pathogenesis of ischemic stroke at high altitude.

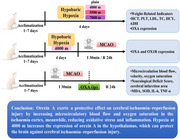

## 1 INTRODUCTION

Stroke is a mounting global health challenge, ranking as the second leading cause of mortality and the third leading cause of disability worldwide. Over the past three decades, there has been a 70% rise in stroke incidence, along with an 85% increase in prevalence and a 43% increase in mortality (Hameed et al., 2024). In China, ischaemic stroke accounts for 79.1% of all stroke cases nationwide (Lu et al., 2020). The epidemiological distribution of ischaemic stroke in China exhibits distinct regional characteristics, marked by high incidence rates in the plateau regions of northwest China, including Tibet and Qinghai (Lu et al., 2020; Yan et al., 2022), highlighting the need for targeted interventions and strategies tailored to these areas.

High altitude is a risk factor for stroke, yet its impact is ambivalent. Residing in areas above 3500 m significantly amplifies the risk of ischaemic stroke, whereas living at an altitude between 1500 and 2500 m appears to confer a protective effect against stroke, likely due to enhanced hypoxia adaptation (Hameed et al., 2024; Ortiz‐Prado et al., 2022). Our team has established a stroke model following 4000 m simulated high‐altitude hypoxia. We observed that, compared to the plain model group, rats subjected to simulated high altitude showed reduced neurological deficit scores and cerebral infarction, along with diminished activation of classical signalling pathways associated with excitotoxic effects (Zhu et al., 2021). Acute mountain sickness (AMS) stands as an important health concern in high altitude regions. Existing literature has implicated that a range of hypoxia‐associated proteins, including orexin A (OXA), hypoxia inducible factor‐1 (HIF‐1), vascular endothelial growth factor (VEGF), and endothelin‐1 (ET‐1), may play a role in the pathophysiology of AMS (Fayazi et al., 2023). Orexin A, primarily secreted by the hypothalamus, regulates feeding behaviour, energy metabolism and cardiovascular functions. Additionally, studies have documented orexin A's neuroprotective effects, shielding neurons against oxidative stress and cerebral ischaemia (Palomba et al., 2020; Xu et al., 2021). It has been reported that the expression of orexin is diminished by both ageing and hypoxia (Adeghate et al., 2020). This leads to the intriguing question: What is the specific impact of altitude on orexin A expression, and does orexin A play a role in the onset and progression of ischaemic stroke in high altitude environment? This issue warrants in‐depth investigation and is the objective of this study.

## 2 METHODS

### 2.1 Ethical approval

This study obtained ethical approval from the Ethics Committee at Medical College of Xizang Minzu University (No. 202206), and all experimental procedures complied with the UK Animals (Scientific Procedures) Act 1986.

### 2.2 Animals

One hundred and fifty‐five specific pathogen free male Sprague–Dawley (SD) rats weighing 200–220 g were procured from the Experimental Animal Centre of Xi'an Jiaotong University (certification number: SCXK (SHAAN) 2018‐001). All rats were acclimated for 1 week under standard laboratory conditions (23 ± 2°C, 50 ± 10% humidity, 12 h light/dark cycle from 07.30 to 19.30 h), with ad libitum access to food and water. All rats that underwent surgical procedures were treated with compound lidocaine cream applied to the wound after surgery to alleviate pain and reduce suffering.

### 2.3 Hypobaric hypoxia imitation, weight and blood related index detection

Fifty‐seven rats were randomly assigned to plain (*n* = 12), 4000 m (*n* = 12), 5500 m (*n* = 15) and 7000 m (*n* = 18) groups. The plain group was placed under normoxic conditions (410 m, Xizang Minzu University, Weicheng District, Xianyang, Shaanxi Province). The hypoxia groups were placed in a hypobaric hypoxia simulation chamber (Xi'an Fukang Air Purification Equipment and Engineering Co., Ltd, Xi'an, China) for 4 days. At 4000 m, the oxygen content is 182.08 g/m^3^, while at 7000 m, 123.16 g/m^3^.

After 4 days of simulated high‐altitude exposure, body weight was recorded in eight rats and compared with pre‐hypoxia values to assess weight loss. Under anaesthesia (3.0–5.0% isoflurane for induction, followed by maintenance at 1.0–2.0%), abdominal circumference and body length were measured to calculate Lee's index (the cube root of bodyweight (g) divided by naso‐anal length (mm)), an index of obesity. Venous blood was collected from the inner canthus for haematocrit and platelet count analysis (Mindray 800 Vet Hematology Analyzer, Mahwah, NJ, USA), followed by abdominal aorta serum collection for antidiuretic hormone (ADH) detection by enzyme‐linked immunosorbent assay (ELISA). Serum from six rats per group was further analysed for low density lipoprotein (LDL), total cholesterol (TC) and homocysteine (HCY) levels. All animals were decapitated under sustained anaesthesia, the whole brain was promptly removed, and hypothalami (located below the thalamus) were dissected for RNA (*n* = 6) or protein (*n* = 6) extraction.

### 2.4 Hypobaric hypoxia combined with middle cerebral artery occlusion modelling

Forty‐five rats were randomly assigned to the sham (*n* = 12), middle cerebral artery occlusion (MCAO) (*n* = 15), and 4000 m + MCAO (*n* = 18) groups. The MCAO model was established using the suture method. After anaesthesia, a specialized suture (Beijing Cinontech Co., Ltd, Beijing, China) was inserted through the common carotid artery into the internal carotid artery (ICA), advancing 18–22 mm from the carotid bifurcation to occlude the origin of the right middle cerebral artery. Rats were subjected to 30 min of ischaemia followed by 24 h of reperfusion. The sham group underwent a similar procedure with a 0.5 cm suture insertion, except without occlusion. For the 4000 m + MCAO group, the MCAO model was established immediately following the completion of the 4000 m simulated hypobaric hypoxia. After MCAO surgery, all animals were raised in a normoxic environment. The compound lidocaine cream was applied to the sutured skin incision for analgesia. When the rats exhibited piloerection, curling up or shivering, additional analgesics were administered again. After 24 h, the rats were euthanized (by exposure to increasing concentrations of CO_2_ followed by decapitation) and then the hypothalami were dissected (*n* = 6 for RNA extraction and *n* = 6 for protein extraction).

### 2.5 Orexin A intervention and related detection

Fifty‐three rats were randomly divided into four groups: sham (*n* = 11), MCAO (*n* = 14), MCAO + NS (normal saline) (*n* = 14), and MCAO + OXA (*n* = 14). Orexin A (cat. no. 003‐30, Phoenix Pharmaceuticals, MA, USA), dissolved in normal saline, was intraperitoneally administered at a dosage of 100 µg/kg (Yang et al., 2017) after inserting the suture. “The MCAO + NS group received an equivalent volume of normal saline, underwent ischaemia for 30 min, and then followed by reperfusion for 24 h.” “Maintaining the same conditions as the second experiment” means that the experimental conditions of ischemia‐reperfusion are the same as those of MCAO after hypoxia. The compound lidocaine cream was applied for analgesia, as described above.

Thirty minutes post‐administration of orexin A, reperfusion was initiated by careful withdrawal of the suture filament, after which the rats were kept under anaesthesia using a breathing mask. The blood flow, velocity and oxygen saturation of microcirculation in the ischaemic cortex were assessed using a laser Doppler (Moor LDI2, Axminster, UK). The detection probe was positioned on the surface of the ischaemic side of the rat's skull, 1 mm posterior to the bregma and 5 mm lateral. Subsequently, the decrease in microcirculatory blood perfusion on the ischaemic side was quantified using laser speckle imaging (Moor FLPI‐2, UK), with the calculation expressed as: (1 − right cerebral blood perfusion/left cerebral blood perfusion) × 100%. In each group, cerebral blood flow was monitored in five rats, with each recording lasting approximately 10–15 min. The rats gradually regained consciousness within about 15 min, with full recovery to normal activity taking 30 min to 1 h. After 24 h of reperfusion, these five rats were re‐anaesthetized using isoflurane (as described previously), and blood was collected from the abdominal aorta for serum separation. The rats were then decapitated, and cortical tissues were harvested. Serum and cortical tissue homogenates were used for superoxide dismutase (SOD) and malondialdehyde (MDA) assays.

The remaining six rats in this experiment underwent neurological function scoring 24 h after reperfusion using the Longa five‐point scoring scale: 0, no neurological deficit; 1, inability to fully extend the contralateral forelimb upon flexion; 2, circling to the contralateral side and tail chasing; 3, body tilting to the contralateral side while walking; and 4, failure to walk spontaneously or unconsciousness.

After the neurological function scoring, the rats were re‐anaesthetized using isoflurane (as described previously). Blood was drawn from abdominal aorta, followed by decapitation. The brains of six rats were then collected and frozen for 30 min before being sectioned into 2 mm coronal slices. These slices were stained with 1% 2,3,5‐triphenyltetrazolium chloride (TTC, Sigma‐Aldrich, St Louis, MO, USA) and incubated at 37°C in the dark for 15–20 min. Viable tissues appeared deep red, while infarcted areas were pale white. The stained slices were fixed in 4% formaldehyde for 24 h at room temperature and photographed using a digital camera. The area of cerebral infarction was measured using ImageJ software (National Institutes of Health, Bethesda, MD, USA). The percentage of infarct area to the total hemispheric area for the second brain slice of each rat was adopted for statistical analysis. In this experiment, serum samples from eight rats per group were selected for ELISA detection of interleukin (IL)‐6 and tumour necrosis factor α (TNF‐α).

### 2.6 ELISA

The supernatant from homogenized cortical tissue or serum from the abdominal aorta blood was used to measure the levels of ADH (E‐EL‐R0522, Elabscience, Wuhan, China), SOD (A001‐3, Nanjing Jiancheng Bioengineering Institute, Nanjing, China), MDA (A003‐1, Nanjing Jiancheng Bioengineering Institute, Nanjing, China), IL‐6 (E‐EL‐R0015, Elabscience, Wuhan, China) and TNF‐α (E‐EL‐R2856, Elabscience, Wuhan, China) using commercial ELISA kits.

### 2.7 Real‐time quantitative polymerase chain reaction analyses

The mRNA was extracted using a TRIzol kit (G3640; Servicebio, Wuhan, China) according to the manufacturer's protocol. One microgram of RNA was reverse‐transcribed into cDNA using the a Servicebio First Strand cDNA Synthesis Kit (G3330; Servicebio). Real‐time quantitative polymerase chain reaction (qPCR) was performed using Servicebio 2 × SYBR Green qPCR Master Mix (G3322). The primers used are shown in Table 1.

**TABLE 1:** Primer used for RT‐qPCR.

Primer	Orientation	Sequence
OXA	Forward	5′‐GACGCTGCTGCTGCTGCTAC‐3′
	Reverse	5′‐CTCCGTGCAACAGTTCGTAGAGAC‐3′
OX1R	Forward	5′‐CCAATCGCACACGGCTCTTCTC‐3′
	Reverse	5′‐CTTCCAGTTCCGCACCAAGGC‐3′
GAPDH	Forward	5′‐CCCTTCATTGACCTCAACTACATG‐3′
	Reverse	5′‐CTTCTCCATGGTGGTGAAGAC‐3′

### 2.8 Western blotting analysis

The hypothalamus tissues were homogenized in RIPA lysis buffer (Beyotime Institute of Biotechnology, Shanghai, China) and protein concentrations were quantified using the bicinchoninic acid (BCA) method (Beyotime Institute of Biotechnology, Shanghai, China). Equal protein amounts (20 mg) from each sample were separated by 10% SDS‐PAGE and transferred to polyvinylidene difluoride membranes at 4°C. Membranes were blocked with 5% non‐fat milk powder in Tris‐buffered saline–Tween‐20 (0.1%) for 2 h at room temperature, then incubated overnight at 4°C with primary antibodies: anti‐Orexin A (PA5‐70436, 1:1000, Thermo Fisher Scientific, Waltham, MA, USA), anti‐OX1R (PA5‐77566, 1:1000, Thermo Fisher Scientific), and β‐actin (3700S, 1:1000, Cell Signaling Technology, Danvers, MA, USA). Following incubation with secondary antibodies for 1 h at room temperature, proteins were visualized using an enhanced chemiluminescence (ECL) system (Beyotime Institute of Biotechnology). Protein band intensities were quantified using ImageJ 2 software.

### 2.9 Statistical analysis

One‐way ANOVA followed by the LSD or Games–Howell multiple comparisons test was used to compare differences among groups. All statistical analyses were carried out using SPSS Statistics 25.0 (IBM Corp., Armonk, NY, USA), and *P* < 0.05 was considered statistically significant. All data are presented as the mean ± SD.

## 3 RESULTS

### 3.1 Acute hypobaric hypoxia reduces weight and obesity

Due to intolerance to hypoxia, three rats died in the 5500 m group and five rats died in the 7000 m group after simulating hypobaric hypoxia.

**FIGURE 1:**
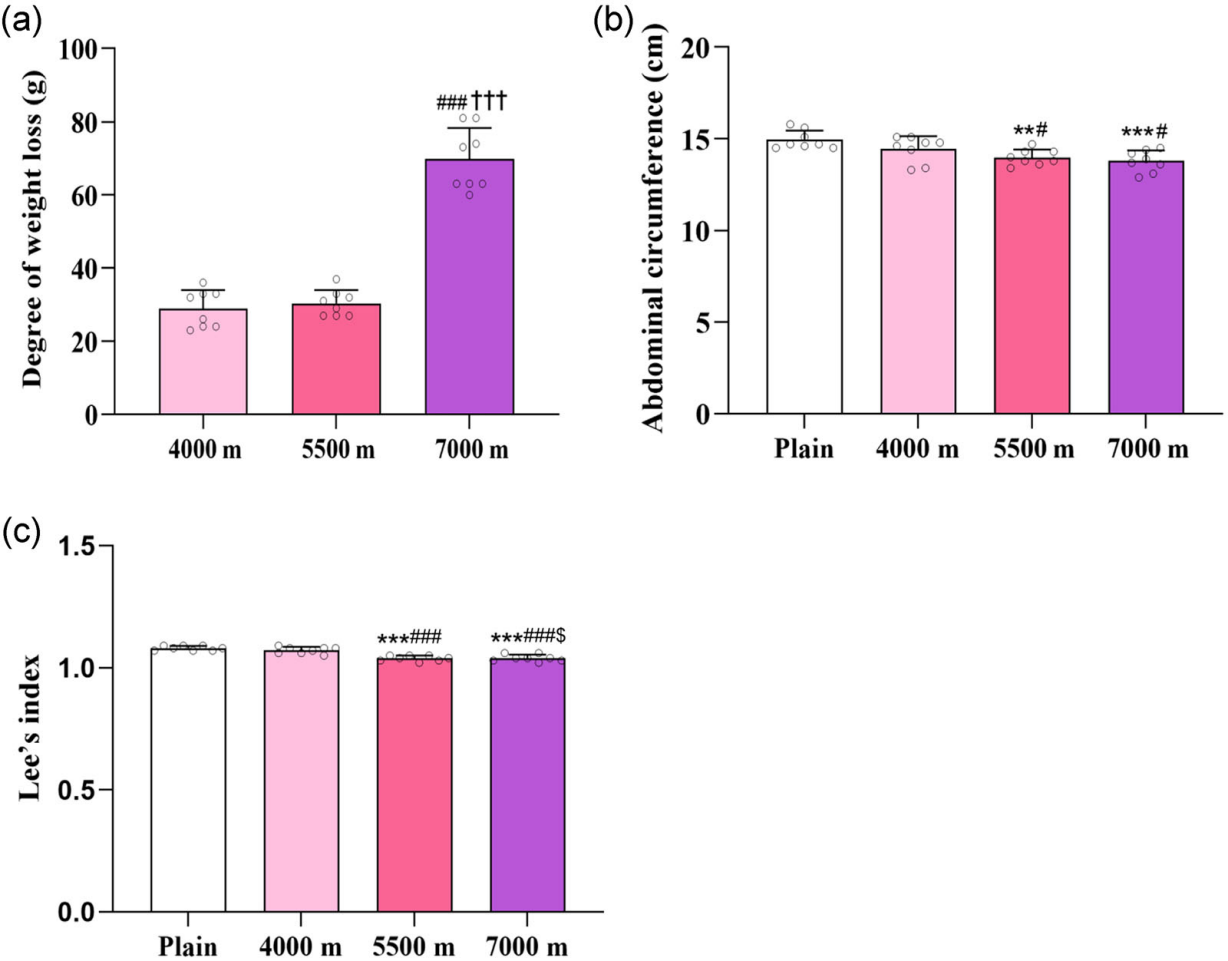
Acute hypobaric hypoxia reduces weight and related indices. (a) Degree of weight loss. (b) Abdominal circumference. (c) Lee's index. ^**^ *P *< 0.01, ^***^ *P *< 0.001, vs. plain group; ^#^ *P *< 0.05, ^###^ *P *< 0.001, vs. 4000 m group; $*P*< 0.05, as compared with 5500 m group. ^†††^ *P *< 0.01, vs. 5500 m group. Data were analysed by one‐way ANOVA followed by LSD (b, c) or Games–Howell (a) multiple comparisons.

In comparison to the 4000 m group (28.88 ± 5.14 g) and 5500 m group (30.38 ± 3.58 g), significant weight loss was observed in the 7000 m group (69.75 ± 8.56 g, *P *< 0.001) (Figure 1a). Compared to the plain group (14.94 ± 0.51 g), abdominal circumference decreased in the 5500 m (13.99 ± 0.43 cm, *P *= 0.0023 <0.01) and 7000 m groups (13.79 ± 0.58 cm, *P *< 0.001) (Figure 1b). Additionally, Lee's index, indicative of obesity, showed a reduction in the 5500 m (1.04 ± 0.01, *P <* 0.001) and 7000 m groups (1.04 ± 0.02, *P <* 0.001) relative to the plain group (1.08 ± 0.01) (Figure 1c).

### 3.2 Acute hypobaric hypoxia increases haematocrit and thrombocyte counts

**FIGURE 2:**
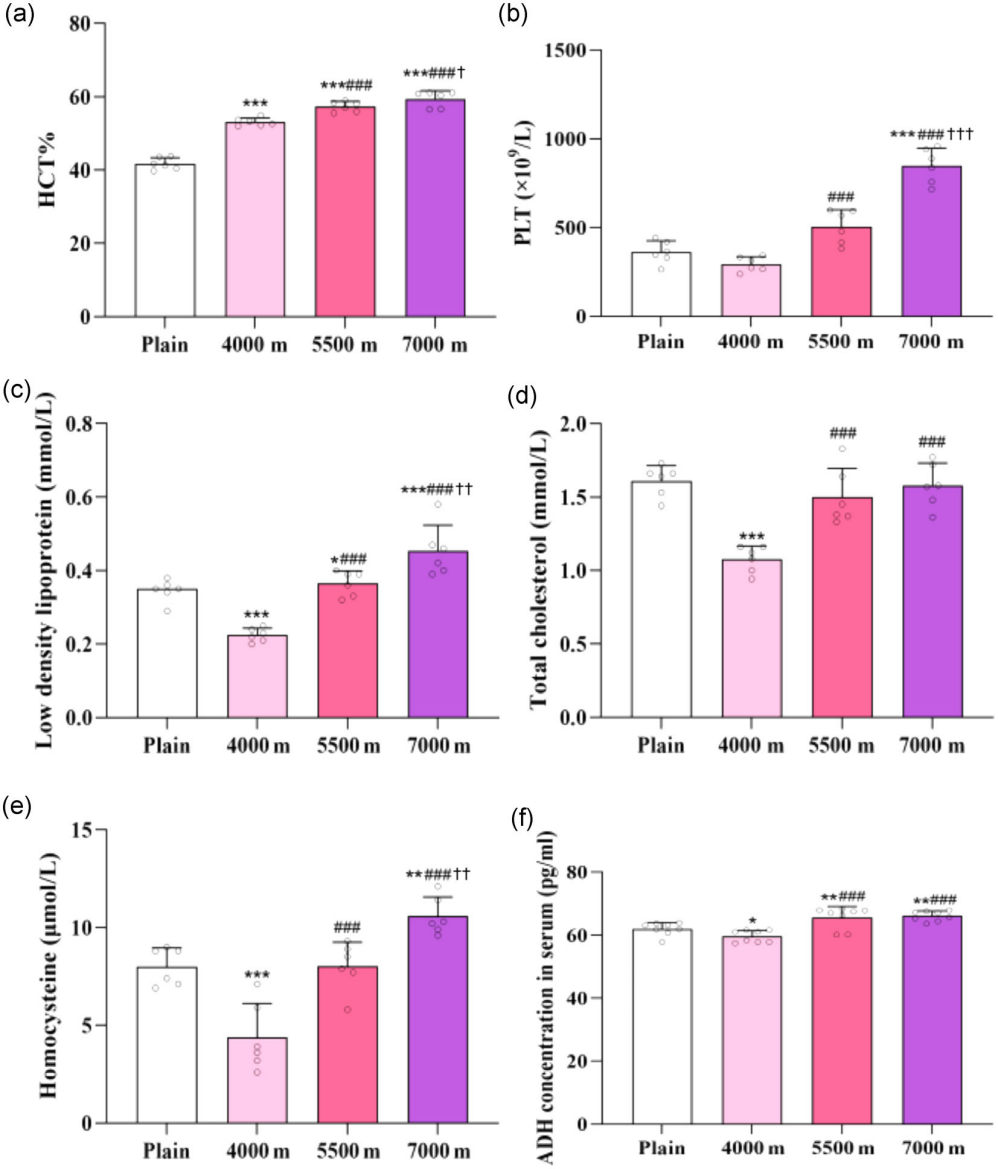
Effects of acute high altitude hypoxia on haematocrit, platelets and metabolism‐related indicators. (a) HCT. (b) PLT. (c) Low density lipoprotein. (d) Total cholesterol. (e) Homocysteine. (F) ADH concentration in serum. ^*^ *P *< 0.05, ^**^ *P *< 0.01, ^***^ *P *< 0.001, vs. plain group; ^###^ *P *< 0.001, vs. 4000 m group; ^†^ *P *< 0.05, ^††^ *P *< 0.01, ^†††^ *P *< 0.001, vs. 5500 m group. Data were analysed by one‐way ANOVA followed by LSD (A, B, C, D, E, F) multiple comparisons.

Haematocrits were notably higher in the 4000 m group (53.19 ± 1.61%, *P *< 0.001), 5500 m group (57.07 ± 1.88%, *P *< 0.001) and 7000 m group (59.12 ± 4.71%, *P *< 0.001) compared to the plain group (41.49 ± 2.57%) (Figure 2a). Platelet counts increased in the 7000 m group (849.00 ± 98.17×10^9^/L, *P *< 0.001) as compared to the plain group (361.33 ± 64.04 × 10^9^/L) Additionally, when compared to the 4000 m group (294.33 ± 41.83 × 10^9^/L), the platelets of rats in the 5500 m group (505.50 ± 94.23 × 10^9^/L, *P *< 0.001) and 7000 m group (*P <* 0.001) also increased (Figure 2b).

### 3.3 Effects of acute hypobaric hypoxia on LDL, total cholesterol and homocysteine levels

Serum levels of LDL were lower in the 4000 m group (0.23 ± 0.02 mmol/L, *P *< 0.001) and higher in the 7000 m group (0.45 ± 0.07 mmol/L, *P *< 0.001) relative to the plain group (0.35 ± 0.03 mmol/L). Additionally, LDL levels were elevated in the 5500 m group (0.37 ± 0.03 mmol/L, *P *< 0.001) and 7000 m group (*P *< 0.001) relative to the 4000 m group (Figure 2c).

Total cholesterol level was lower in the 4000 m group (1.08 ± 0.09 mmol/L, *P <* 0.001) than in the plain group (1.61 ± 0.11 mmol/L). Compared to the 4000 m group, total cholesterol levels were higher in both the 5500 m group (1.50 ± 0.20 mmol/L, *P <* 0.001) and 7000 m group (1.58 ± 0.15 mmol/L, *P <* 0.001) (Figure 2d).

Homocysteine levels were lower in the 4000 m group (4.38 ± 1.74 µmol/L, *P *< 0.001) and higher in the 7000 m group (10.58 ± 0.96 µmol/L, *P *= 0.0021 <0.01) relative to the plain group (8.00 ± 0.97 µmol/L). Compared to the 4000 m group, homocysteine levels were elevated in the 5500 m group (8.02 ± 1.24 µmol/L, *P *< 0.001) and 7000 m group (*P *< 0.001) (Figure 2e).

### 3.4 Effects of acute hypobaric hypoxia on ADH levels

Serum ADH levels were lower in 4000 m group (59.60 ± 1.87 pg/ml, *P *= 0.0478 *<* 0.05), while higher in the 5500 m group (65.65 ± 3.46 pg/ml, *P *= 0.0045 <0.01) and 7000 m group (66.12 ± 1.58 pg/ml, *P *= 0.0016 <0.01) relative to the plain group (62.03 ± 2.02 pg/ml); compared to the 4000 m group, serum ADH levels were markedly higher in the 5500 m group (*P *< 0.001) and 7000 m group (*P *< 0.001) (Figure 2f).

### 3.5 Effects of acute hypobaric hypoxia on OXA expression

**FIGURE 3:**
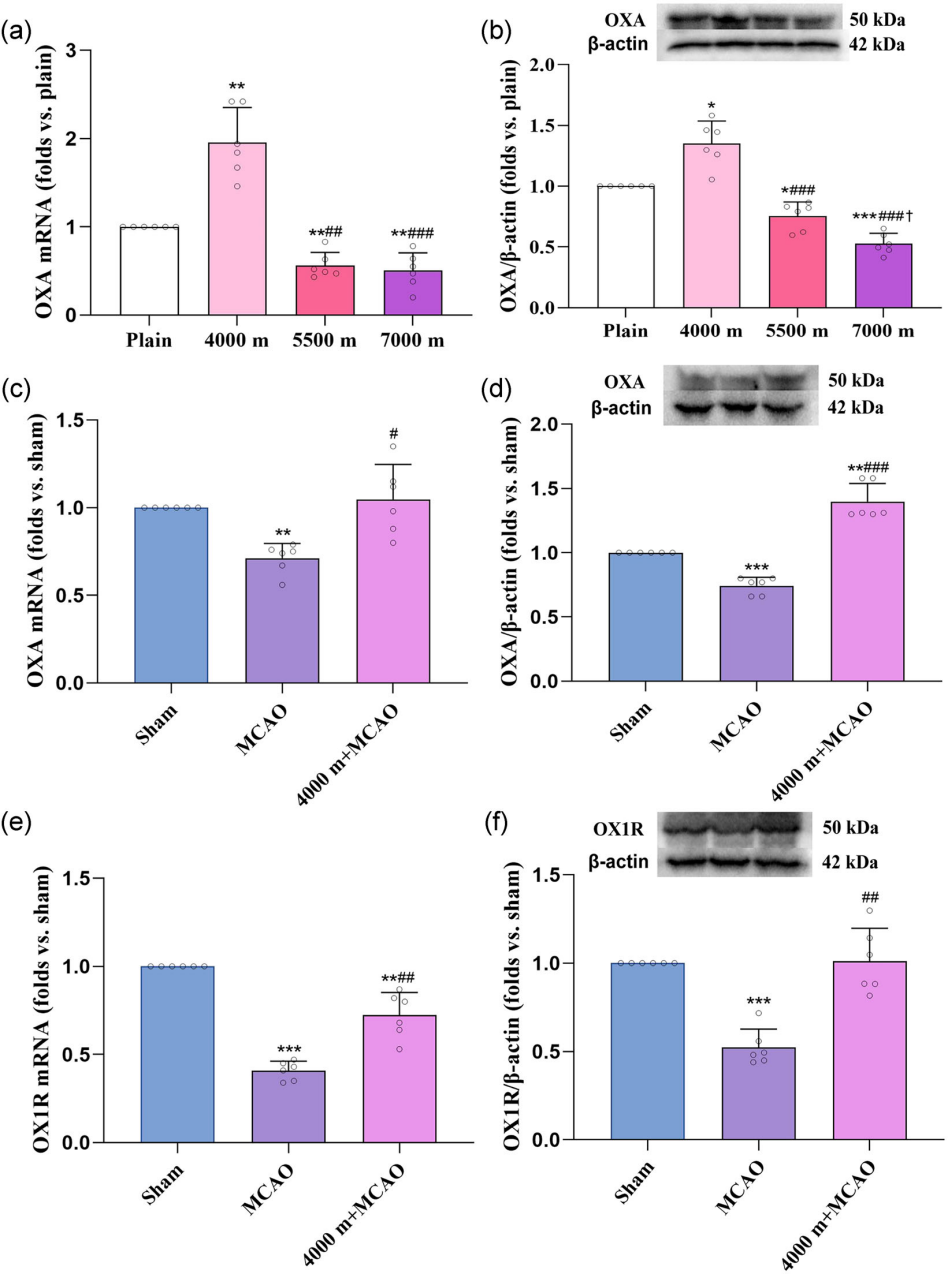
Effects of acute high altitude hypoxia and cerebral ischaemia–reperfusion on the expression of OXA and OX1R. (a, c) OXA gene expression. (b, d) OXA protein expression. (e) OX1R gene expression. (f) OX1R protein expression. For (a, b), ^*^ *P *< 0.05, ^**^ *P *< 0.01, ^***^ *P *< 0.001, vs. plain group; ^##^ *P *< 0.01, ^###^ *P *< 0.001, vs. 4000 m group; ^†^ *P *< 0.05, vs. 5500 m group. For (c–f), ^**^ *P *< 0.01, ^***^ *P *< 0.001, vs. sham group; ^#^ *P *< 0.05, ^##^ *P *< 0.01, vs. MCAO group. Data were analysed by one‐way ANOVA followed by Games–Howell (a, b, c, d, e, f) multiple comparisons.

OXA gene expression was upregulated in the 4000 m group (1.96 ± 0.39, *P *< 0.01) and downregulated in the 5500 m group (0.56 ± 0.15, *P *< 0.01) and 7000 m group (0.50 ± 0.20, *P *< 0.01) relative to the plain group (1.00 ± 0.00) (Figure 3a). Consistently, OXA protein expression was elevated in the 4000 m group (1.35 ± 0.19, *P *< 0.05) and reduced in the 5500 m group (0.75 ± 0.11, *P *< 0.05) and 7000 m group (0.53 ± 0.09, *P *< 0.001) compared to the plain group (1.00 ± 0.00) (Figure 3b).

### 3.6 Effects of acute hypobaric hypoxic preconditioning on OXA and OXR1 expression in rats with cerebral ischaemia–reperfusion injury

In the experiment simulating cerebral ischaemia–reperfusion after high altitude hypoxia exposure, no mortality was observed in the sham group, whereas three rats died in the MCAO group, and five rats died in the 4000 m+MCAO group. This increased mortality is attributed to the inherent trauma associated with MCAO modelling, which poses a significant threat to the animals' survival. The combination of MCAO with high altitude hypoxia further exacerbates the mortality rate.

OXA gene expression was decreased in the MCAO group (0.71 ± 0.08, *P *= 0.0009 <0.01) relative to the sham group (1.00 ± 0.00); while compared with MCAO group, OXA gene expression was increased in the 4000 m+MCAO group (1.05 ± 0.20, *P *= 0.0180 *<*0.05) (Figure 3c).

OXA protein expression was decreased in the MCAO group (0.74 ± 0.06, *P *< 0.001) and increased in the 4000 m+MCAO group (1.40 ± 0.14, *P *< 0.01) when compared to the sham group (Figure 3d).

Compared with the sham group (1.00 ± 0.00), OX1R gene and protein expression were decreased in MCAO group (0.41 ± 0.05, *P <* 0.001; 0.52 ± 0.10, *P <* 0.001); as compared with the MCAO group, OX1R gene and protein expression were increased the 4000 m+MCAO group (0.72 ± 0.13, *P *= 0.0024 *<*0.01; 1.01 ± 0.19, *P *= 0.0013 *<*0.01) (Figure 3e, f).

### 3.7 Neuroprotective effects of orexin A on cerebral ischaemia–reperfusion injury

In the experiment with orexin A intervention, mortality was observed across different groups: three rats died in the MCAO group, two in the MCAO+NS group, and two in the MCAO+OXA group. The primary cause of death was intolerance to the MCAO surgery.

#### 3.7.1 Orexin A increased microcirculation blood flow, velocity and oxygen saturation in the ischaemic cortex of rats

**FIGURE 4:**
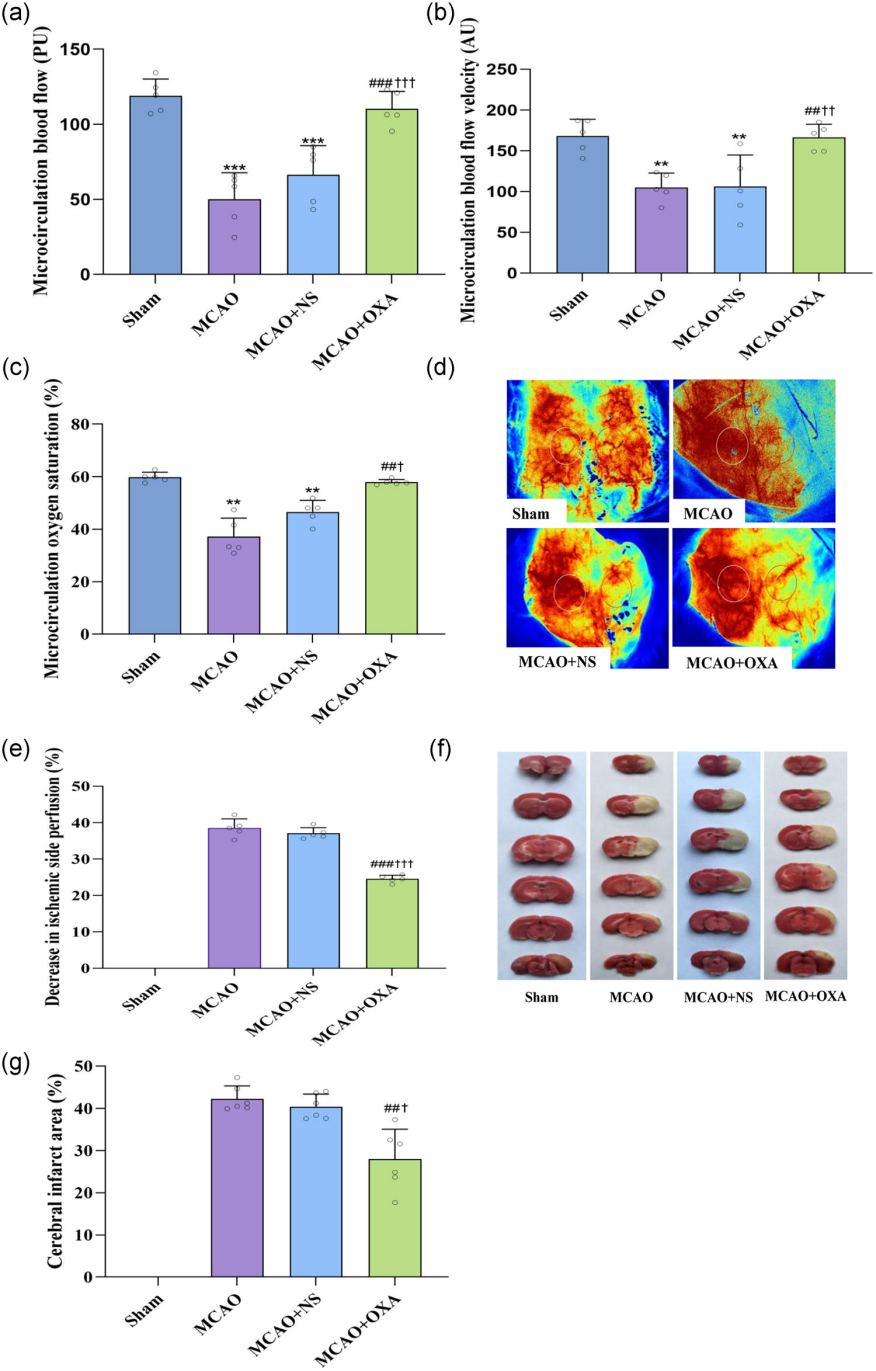
OXA reduced cerebral infarction and increased microcirculation perfusion of the ischaemic cortex. (a) Microcirculation blood flow. (b) Microcirculation blood flow velocity. (c) Microcirculation oxygen saturation. (d) Laser speckle. (e) Decrease of microcirculation perfusion in ischaemic cortex. (f) TTC staining. (g) Statistics of cerebral infarction area. ^**^ *P *< 0.01, ^***^ *P *< 0.001, vs. sham group; ^##^ *P <* 0.01, ^###^ *P *< 0.001, vs. MCAO group; ^†^ *P *< 0.05, ^††^ *P *< 0.01, ^†††^ *P *< 0.001, vs. MCAO + NS. Data were analysed by one‐way ANOVA followed by LSD (a, b,) or Games–Howell (c, e, g) multiple comparisons.

In comparison to the sham group (118.96 ± 11.23 Perfuion Units (PU)), microcirculation blood flow in the ischaemic cortex of rats was lower in the MCAO group (50.07 ± 17.72 PU, *P *< 0.001) and the MCAO+NS group (66.55 ± 19.24 PU, *P <* 0.001); while as compared with the MCAO group, the microcirculation blood flow was higher in the MCAO+OXA group (110.45 ± 11.49 PU (perfuion units), *P <* 0.001) (Figure 4a).

Similarly, microcirculation blood flow velocity in the ischaemic cortex was significantly decreased in the MCAO group (105.15 ± 17.45 AU, *P *= 0.0011 <0.01) and MCAO+NS group (106.07 ± 38.79 AU, *P *= 0.0013 <0.01) compared to the sham group (168.18 ± 20.57 AU); compared with the MCAO group, the velocity increased in the MCAO+OXA group (166.24 ± 16.27 AU, *P *= 0.0014 *<*0.01) (Figure 4b).

Regarding microcirculation oxygen saturation in the ischaemic cortex, while compared with the sham group (59.77 ± 1.88%), the microcirculation oxygen saturation in the ischaemic cortex of rats was lower in MCAO group (37.21 ± 7.01%, *P *= 0.0050 <0.01) and MCAO+NS group (46.58 ± 4.38%, *P *= 0.0050, <0.01). In contrast, the MCAO+OXA group (57.87 ± 1.01%, *P *= 0.0087, *<*0.01) displayed an increase in microcirculation oxygen saturation compared to the MCAO group (Figure 4c).

The decrease in perfusion on the ischaemic cortex were lower in the MCAO+OXA group (24.52 ± 1.02%) as compared with the MCAO group (38.54 ± 2.51%, *P *< 0.001) and the MCAO+NS group (37.07 ± 1.56%, *P *< 0.001) (Figure 4d, e).

#### 3.7.2 Orexin A decreased neurological deficit scores in rats with cerebral ischaemia–reperfusion injury

Neurological deficit scores were lower in the MCAO+OXA group (1.17 ± 0.41) compared to both MCAO (2.00 ± 0.63, *P *= 0.0106 <0.05) and MCAO+NS groups (1.83 ± 0.41, *P = *0.0338 <0.05) (Table 2).

**TABLE 2:** Neurological deficit score at 24 h after cerebral ischaemia–reperfusion.

	1	2	3	4	5	6	Means ± SD.
Sham	0	0	0	0	0	0	0.00 ± 0.00
MCAO	2	2	3	2	2	1	2.00 ± 0.63
MCAO + NS	2	2	2	2	2	1	1.83 ± 0.41
MCAO + OXA	2	1	1	1	1	1	1.17 ± 0.41^#^ ^†^

^#^ *P <* 0.05, versus MCAO group; ^†^ *P <* 0.05, versus MCAO + NS group. Data were analysed by one‐way ANOVA followed by LSD.

#### 3.7.3 Orexin A reduced cerebral infarction in rats with cerebral ischaemia–reperfusion injury

Cerebral infarction was reduced in the MCAO+OXA group (27.95 ± 7.13%, *P *= 0.0072 <0.01; *P *= 0.0146 <0.05) compared to the MCAO group (42.29 ± 3.02%) and MCAO+NS group (40.40 ± 2.96%) (Figure 4f, g).

#### 3.7.4 Orexin A attenuated oxidative stress and inflammation

##### Orexin A suppresses oxidative stress in rats with cerebral ischaemia–reperfusion injury

**FIGURE 5:**
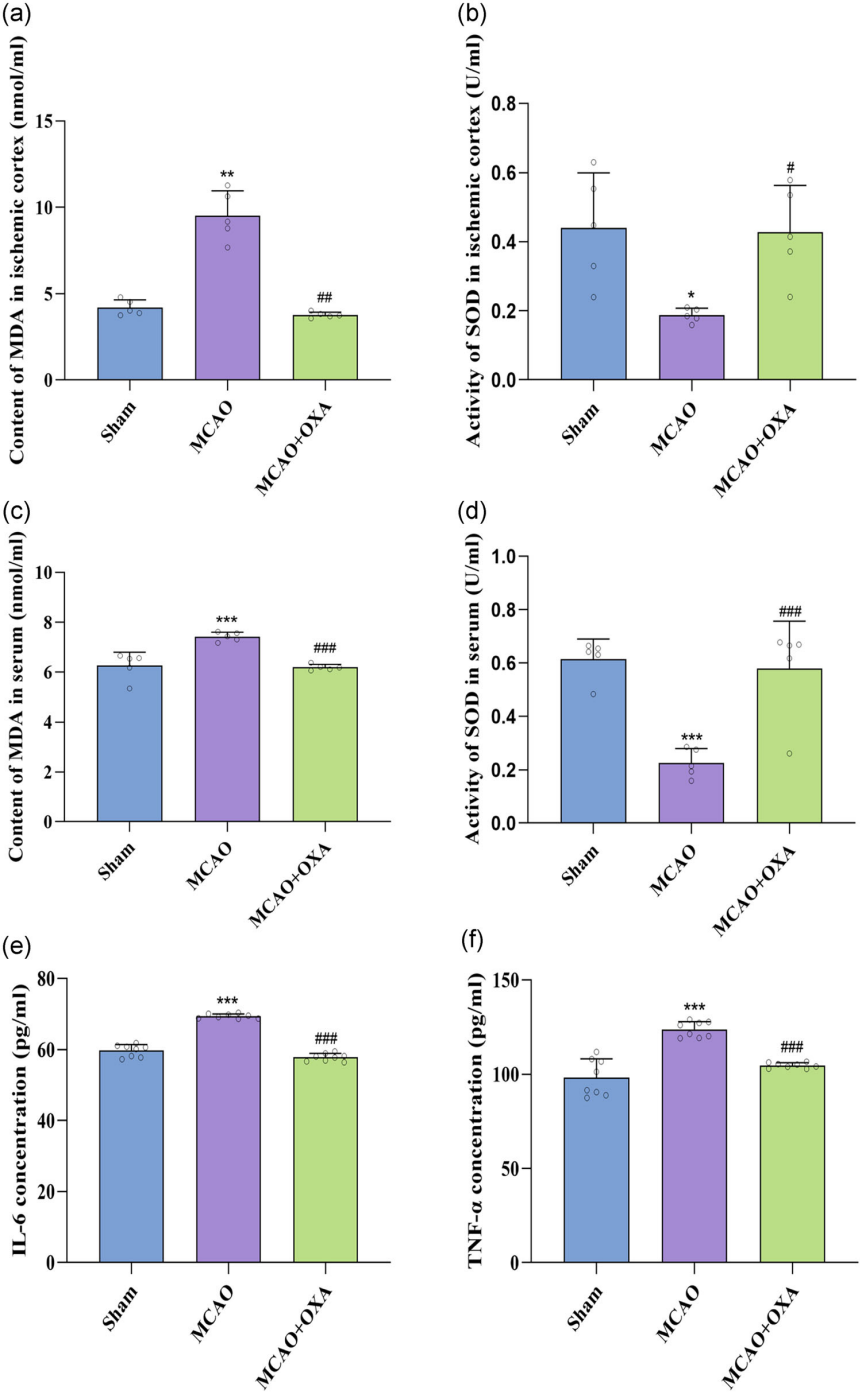
OXA decreased oxidative stress and inflammation. (a) Content of MDA in ischaemic cortex. (b) Vitality of SOD in ischaemic cortex. (c) Content of MDA in serum. (d) Vitality of SOD in serum. (e) IL‐6 concentration. (f) TNF‐α concentration. ^*^ *P *< 0.05, ^**^ *P *< 0.01, ^***^ *P *< 0.001, vs. sham group; ^#^ *P *< 0.05, ^##^ *P *< 0.01, ^###^ *P *< 0.001, vs. MCAO group. Data were analysed by one‐way ANOVA followed by LSD (c, d) or Games–Howell (a, b, e, f) multiple comparisons.

In the ischaemic cortex, MDA content was significantly elevated in the MCAO group (9.51 ± 1.45 nmol/ml, *P *= 0.0016 <0.01) than in the sham group (4.19 ± 0.44 nmol/ml). However, orexin A treatment (MCAO+OXA) significantly reduced MDA content in the ischaemic cortex (3.77 ± 0.17 nmol/ml, *P *= 0.0018 <0.01) compared to the MCAO group (Figure 5a). Similarly, serum MDA content was also increased in the MCAO group (7.41 ± 0.18 nmol/ml, *P *< 0.001) versus the sham group (6.25 ± 0.54 nmol/ml), but was attenuated in the MCAO+OXA group (6.19 ± 0.12 nmol/ml, *P *< 0.001) as compared with the MCAO group (Figure 5c).

Regarding SOD activity, a significant decrease was noted in the ischaemic cortex of the MCAO group (0.19 ± 0.02 U/ml *P *= 0.0495 < 0.05) compared to the sham group (0.44 ± 0.16 U/ml). Conversely, the MCAO+OXA group showed a notable increase in SOD activity in the ischaemic cortex (0.43 ± 0.13 U/ml, *P *= 0.0334 <0.05) compared to the MCAO group (Figure 5b). Similarly, serum SOD activity was reduced in the MCAO group (0.23 ± 0.054 U/ml, *P *< 0.001) compared to the sham group (0.61 ± 0.074 U/ml), but was significantly enhanced in the MCAO+OXA group (0.58 ± 0.18 U/ml, *P *< 0.001) relative to MCAO group (Figure 5d).

##### Orexin A mitigated inflammation in rats with cerebral ischaemia–reperfusion injury

Serum IL‐6 level was significantly elevated in the MCAO group (69.38 ± 0.65 pg/ml, *P *< 0.001) compared with the sham group (59.70 ± 1.72 pg/ml). Notably, the MCAO+OXA group demonstrated a significant reduction in serum IL‐6 level (57.79 ± 1.08 pg/ml, *P *< 0.001) compared to the MCAO group (Figure 5e).

Similarly, serum TNF‐α level was significantly increased in the MCAO group (123.73 ± 4.16 pg/ml, *P *< 0.001) compared to the sham group (98.29 ± 9.81 pg/ml). Treatment with orexin A (MCAO+OXA) reduced serum TNF‐α level (104.64 ± 1.45 pg/ml, *P *< 0.001) relative to the MCAO group (Figure 5f).

## 4 DISCUSSION

Air pollution, climate change, gut microbiota, high altitude and systemic infections have been identified as significant risk factors for the development of stroke (Hameed et al., 2024). Notably, around 5.7% of the global population resides at altitudes above 1500 m (Ortiz‐Prado et al., 2022). Three independent studies from the United States, Austria and Ecuador consistently show that residing at medium to high altitude (below 3500 m) is associated with reduced mortality rates from ischaemic heart disease and stroke (Hameed et al., 2024). Particularly, the protective effect is more significant within the 2000–3500 m altitude range (Hameed et al., 2024; Ortiz‐Prado et al., 2021). Furthermore, an observational study in the sub‐Himalayan region of India reported a lower incidence of stroke among hospitalized patients at a medium altitude of 2000 m (Hameed et al., 2024). However, it is important to note that above 3500–4000 m, the risk of stroke escalates, particularly for non‐adapted populations with acute exposure (Ortiz‐Prado et al., 2022). These findings underscore the intricate relationship between altitude and stroke risk, warranting further research to uncover the mechanisms involved and guide the development of targeted interventions. An epidemiological study on stroke incidence in Tibet revealed that stroke patients are getting younger at high altitude, with increased red blood cell count and homocysteine level as independent risk factors (Lu et al., 2020). The rise in red blood cells may be due to the chronic hypoxic conditions, while elevated homocysteine level could be linked to dietary patterns that emphasize meat consumption over vegetables (Liu et al., 2021; Lu et al., 2020). High‐altitude hypoxia initiates a series of physiological responses, such as compensatory hyperventilation, tachycardia, vasoconstriction, erythrocytosis, increased blood viscosity and dehydration, which in turn raise the risk of thromboembolic complications (Dunn et al., 2012; Hameed et al., 2024; Ortiz‐Prado et al., 2022). Our current investigation has further highlighted a positive correlation between increasing altitude and elevation of haematocrit and platelet counts. This comprehensive set of changes contributes to heightened blood viscosity, a critical factor in the increased incidence of ischaemic stroke. Concurrently, we observed a parallel increase in ADH levels with rising altitude. We hypothesize that this elevation in ADH may induce vasoconstriction and a pressor effect, thus becoming an additional factor that could precipitate or exacerbate cerebrovascular disease. Our findings underscore the intricate interplay between altitude, physiological adaptations and the risk of cerebrovascular complications.

Homocysteine emerges as an independent and graded predictor of cardiovascular and cerebrovascular events, including ischaemic stroke and myocardial infarction, in both plain and high altitude. The primary mechanisms by which elevated homocysteine contributes to vascular events involve impaired vascular endothelial function, augmented oxidative stress, dysregulated lipid metabolism and thrombosis (Liu et al., 2021; Pinzon et al., 2023; Yan et al., 2022; Yuan et al., 2021). Our current investigation reveals a pronounced elevation in homocysteine levels with rising altitude. While previous studies have implicated a meaty diet as a contributing factor to elevated homocysteine, this study highlights that, beyond dietary factors, hypoxia at high altitudes is a crucial factor in elevating homocysteine concentration.

Ischaemic stroke patients in plateau regions, in addition to having higher homocysteine levels, also exhibited significantly elevated triglyceride concentrations, atherosclerosis indices and plaque formation compared to those residing in plain areas. Paradoxically, while some reports suggest a lower prevalence of hyperlipidaemia among high‐altitude dwellers (Liu et al., 2021; Ortiz‐Prado et al., 2022), our study indicates a progressive increase in LDL and total cholesterol levels with altitude, potentially underpinning the incidence of ischaemic stroke to a certain degree. It is recognized that low‐density lipoprotein cholesterol (LDL‐C) is a major risk factor for cardiovascular disease, for LDL‐C is prone to oxidation, and oxidized LDL‐C can trigger inflammation, leading to vascular damage and atherosclerosis. Maintaining optimal LDL‐C levels in young and middle‐aged individuals can mitigate the lifetime risk of developing atherosclerotic cardiovascular disease (Zhang et al., 2021). In a parallel group trial conducted in France and South Korea, ischaemic stroke or TIA patients with atherosclerosis were treated with statins, ezetimibe or a combination of both, revealing that patients with LDL‐C levels below 70 mg/dL had a lower risk of subsequent cardiovascular events than those with LDL‐C levels between 90 and 110 mg/dL (Amarenco et al., 2020). Generally, at altitudes exceeding 4000 m, there is a tendency for plasma homocysteine, LDL and cholesterol levels to increase with altitude, which are all significant risk factors for the occurrence of high altitude cerebral stroke. Under simulated conditions of 4000 m altitude, lower levels of these three important indicators have been observed, potentially accounting for the relatively mild ischaemic stroke injury at this altitude. However, the specific reasons for the reduction in these indicators at 4000 m warrant further investigation.

Orexin, a vital neuropeptide, is comprised of two distinct subtypes: orexin A and orexin B, as well as two types of receptors, the orexin type 1 receptor (OX1R) and orexin type 2 receptor (OX2R). Orexins and their receptors are widely distributed throughout the central nervous system and peripheral tissues, indicating their extensive effects (Adeghate et al., 2020; Berhe et al., 2020; Kuwaki, 2021; Soejima et al., 2023). Orexin plays a crucial role in orchestrating various functional activities in mammals, including cardiovascular regulation, food consumption, metabolic processes, wakefulness and sleep (Berhe et al., 2020; Fayazi et al., 2023). Following hypoxic exposure, a notable decrease in orexin A gene expression in the hypothalamus of newborn pigs has been documented, suggesting a potential link to hypoxia‐induced behaviours such as narcolepsy and anorexia at high altitudes (Du et al., 2016). Notably, despite limited literature on the impact of altitude on orexin expression, our study has yielded intriguing results. Specifically, orexin A expression was found to increase at an altitude of 4000 m, with a significant decrease beyond this elevation. Given orexin's vital role in energy metabolism and appetite regulation, the decrease in orexin A expression at higher altitude offers a plausible explanation for the exacerbated weight loss observed with increasing altitude. It has been reported that significant weight loss occurs at altitude exceeding 5000 m (Santangelo et al., 2024), a finding consistent with our results. In addition to weight reduction, we also noted marked decreases in abdominal circumference and Lee's index, underscoring the comprehensive effects of altitude on body dimensions and composition.

In addition to regulating the physiological functions mentioned, orexin has been recognized for its neuroprotective effects on cerebral ischaemia–reperfusion injury by mitigating inflammation and oxidative stress, thereby effectively reducing apoptosis and autophagy (Xu et al., 2021, 2021; Zhao et al., 2021). To delve deeper into the role of orexin in high altitude ischaemic stroke, we initially employed Sprague–Dawley rats to simulate high‐altitude hypoxic conditions. Subsequently, we established a MCAO model to replicate the pathological processes of ischaemic stroke. Our results indicated that, in comparison to the plain MCAO group, rats in the MCAO group exposure to simulated 4000 m altitude hypoxia exhibited an upregulation of orexin A and its receptor OX1R. In 2021, we confirmed that these rats also demonstrated decreased neurological deficit scores and a reduced cerebral infarction area (Zhu et al., 2021). Together, these findings suggest that preconditioning rats to simulated 4000 m altitude enhanced their hypoxic adaptation, which in turn alleviated the severity of cerebral ischaemia–reperfusion injury. Notably, this enhanced adaptation was closely associated with the increased expression of orexin A, highlighting its pivotal role in protecting against the detrimental effects of high altitude ischaemic stroke.

We then conducted an in‐depth investigation into the protective effect and mechanism of orexin A in mitigating cerebral ischaemia–reperfusion injury via exogenous administration. Our findings revealed that orexin A intervention significantly alleviated the severe reduction in cortical blood flow during the reperfusion phase, while also enhancing blood flow velocity and oxygen saturation. Adequate blood flow and oxygen supply are crucial for the survival of neurons (Ortiz‐Prado et al., 2010). The intervention of orexin A not only diminished the severity of cerebral ischaemia–reperfusion injury but also positively contributed to the recovery of neuronal function subsequent to ischaemia. A prior study has demonstrated that orexin A is capable of crossing the blood–brain barrier (BBB) (Kastin & Akerstrom, 1999). In our current investigation, orexin A was administered intraperitoneally, leading us to hypothesize that it can traverse the BBB to exert neuroprotective effects on neurons. Furthermore, it has been reported that OX1R and OX2R are expressed on the vascular endothelium (Kim et al., 2015). Our observations indicate that orexin A enhances cerebral blood flow during ischaemia–reperfusion, suggesting that it may also exert peripheral effects. Based on the HIF‐1α–ET‐1/nitric oxide synthase signalling pathway, we have verified the central protective effect of orexin A on cerebral ischaemia–reperfusion injury in another study (Zhu et al., 2024). In this study, we observed the protective effect of orexin A on cerebral ischaemia–reperfusion injury. Whether its primary action is exerted on central neurons via BBB or if it works together with peripheral effects remains to be further verified.

In recent years, research into the pathophysiological mechanisms has established that acute ischaemic stroke (AIS) is a thrombo‐inflammatory disease (Liu et al., 2021). Hypoxia‐induced inflammation at high altitudes may exacerbate the inflammatory cascade, promoting thrombosis and resulting in more severe high‐altitude stroke (Liu et al., 2021). Patients with anterior circulation AIS in plateau regions exhibit pronounced inflammatory responses, large infarct volumes, more severe neurological symptoms and a poor prognosis (Yan et al., 2022). IL‐6, a key cytokine in the inflammation response following cerebral ischaemia, when overexpressed, exacerbates tissue inflammation and acts as a prognostic indicator in stroke (Wan et al., 2021). Our current investigation uncovered that orexin A administration suppressed the expression of IL‐6 and TNF‐α, while simultaneously elevating SOD levels and decreasing MDA levels. These findings align with previous reports that highlight orexin's anti‐inflammatory, antioxidant and anti‐apoptotic properties (Jo et al., 2022; Xu et al., 2021), thereby elucidating the mechanisms underlying mild brain injury observed in rats subjected to 4000 m of hypoxia in the MCAO model.

### 4.1 Conclusion

Given the emerging trend of ischaemic stroke affecting younger individuals at high altitudes, it is crucial to proactively implement preventative and therapeutic strategies for cerebrovascular diseases in plateau regions. The escalation of haematocrit, cholesterol, low density lipoprotein and homocysteine levels with increasing altitude underscores their pivotal role as risk factors for high altitude ischaemic stroke. Notably, exposure to lower altitudes (below 4000 m) promotes brain adaptation to hypoxia, thereby mitigating the severity of cerebral ischaemic injury following hypoxic episodes. This protective effect is attributed to the enhanced expression of orexin A at these altitudes, conferring neuroprotection. Despite the limited research on the protective mechanisms of orexin against cerebral ischaemia–reperfusion injury, the current findings offer valuable insights into the variability in ischaemic stroke incidence and pathological damage at different altitudes. Furthermore, these findings suggest the potential of orexin A as a promising therapeutic target for the prevention and management of cerebrovascular diseases at high altitudes. The study, limited to male rats, has constraints in applying results to all stroke patients. We intend to include female rats in future studies for broader applicability and, if possible, confirm our results with human studies.

## AUTHOR CONTRIBUTIONS

Minxia Zhu: Conceptualization; data curation; funding acquisition; project administration; supervision; writing – original draft; writing – review & editing. Xu Guo: Formal analysis; methodology; investigation. Jing Guo, Zhaojun Zhang, Kexin Zhang, Yaqi Lei, Yuhe Wang and Haoshuang Zhan: Methodology; investigation. All authors have read and approved the final version of this manuscript and agree to be accountable for all aspects of the work in ensuring that questions related to the accuracy or integrity of any part of the work are appropriately investigated and resolved. All persons designated as authors qualify for authorship, and all those who qualify for authorship are listed.

## CONFLICT OF INTEREST

None declared.

## Data Availability

All the data generated or analysed during this study are included in this manuscript.
